# Tumor-infiltrating lymphocytes predict cutaneous melanoma survival

**DOI:** 10.1186/1479-5876-13-S1-O9

**Published:** 2015-01-15

**Authors:** Cristina Fortes, Simona Mastroeni, Thomas Manooranparanpampil, Francesca Passarelli, Alba Zappalà, Claudia Marino, Nicoletta Russo, Paola Michelozzi

**Affiliations:** 1 Epidemiology Unit, IDI-IRCCS, Rome, Italy; 2 Pathology Unit, IDI-IRCCS, Rome, Italy; 3 Oncology Unit , IDI-IRCCS, Rome, Italy; 4 Department of Epidemiology of the Regional Health Service, Rome, Italy; 5 Medical Direction, IDI-IRCCS, Rome, Italy

## Background

Tumor-infiltrating lymphocytes (TILs) is considered a manifestation of the host immune response to tumor, but the role of TILs on melanoma mortality is controversial. Therefore, the aim of this study was to investigate the role of TILs on melanoma mortality, controlling for all known histological prognostic parameters.

## Materials and methods

We conducted a 10-year cohort study among 4143 patients from the same geographic area (Lazio) with primary cutaneous melanoma diagnosed between January 1998 and December 2008. Survival probability was determined by Kaplan–Meier estimates, and prognostic factors were evaluated by multivariate analysis (Cox proportional hazards model).

## Results

Survival decreased with increasing age (P for trend < 0.001) and Breslow thickness (P for trend < 0.001). In the multivariate Cox model, the presence of high levels of tumour infiltrating immune cells in primary invasive melanomas was associated with lower risk of melanoma death (RR: 0.32; 95%CI:0.13-0.82, P for trend <0.001), after controlling for sex, age, breslow thickness, histological type, mitotic rate and ulceration.

## Conclusions

These results suggest that immune microenvironment affects melanoma survival. Understanding differences in survival across distinct subgroups of melanoma patients may help choosing types of therapy.

